# Deletion of the Homocysteine Thiolactone Detoxifying Enzyme Bleomycin Hydrolase, in Mice, Causes Memory and Neurological Deficits and Worsens Alzheimer’s Disease-Related Behavioral and Biochemical Traits in the 5xFAD Model of Alzheimer’s Disease

**DOI:** 10.3233/JAD-230578

**Published:** 2023-10-10

**Authors:** Łukasz Witucki, Kamila Borowczyk, Joanna Suszyńska-Zajczyk, Ewelina Warzych, Piotr Pawlak, Hieronim Jakubowski

**Affiliations:** Department of Biochemistry and Biotechnology, Poznań University of Life Sciences, Poznań, Poland; b Department of Genetics and Animal Breeding, Poznań University of Life Sciences, Poznań, Poland; c Department of Microbiology, Biochemistry and Molecular Genetics, Rutgers University, New Jersey Medical School, International Center for Public Health, Newark, NJ, USA

**Keywords:** Alzheimer’s disease, amyloid-β protein precursor, autophagy, bleomycin hydrolase, *Blmh*^-/-^*5xFAD* mouse, H4K20me1, homocysteine thiolactone, mTOR, N2a-APPswe mouse neuroblastoma cells, Phf8

## Abstract

**Background:**

Bleomycin hydrolase (BLMH), a homocysteine (Hcy)-thiolactone detoxifying enzyme, is attenuated in Alzheimer’s disease (AD) brains. Blmh loss causes astrogliosis in mice while the loss of histone demethylase Phf8, which controls mTOR signaling, causes neuropathy in mice and humans.

**Objective:**

To examine how *Blmh* gene deletion affects the Phf8/H4K20me1/mTOR/autophagy pathway, amyloid-β (Aβ) accumulation, and cognitive/neuromotor performance in mice.

**Methods:**

We generated a new mouse model of AD, the *Blmh*^-/-^5xFAD mouse. Behavioral assessments were conducted by cognitive/neuromotor testing. *Blmh* and *Phf8* genes were silenced in mouse neuroblastoma N2a-APPswe cells by RNA interference. mTOR- and autophagy-related proteins, and AβPP were quantified by western blotting and the corresponding mRNAs by RT-qPCR. Aβ was quantified by western blotting (brains) and by confocal microscopy (cells).

**Results:**

Behavioral testing showed cognitive/neuromotor deficits in *Blmh*^-/-^ and *Blmh*^-/-^5xFAD mice. Phf8 was transcriptionally downregulated in *Blmh*^-/-^ and *Blmh*^-/-^5xFAD brains. H4K20me1, mTOR, phospho-mTOR, and AβPP were upregulated while autophagy markers Becn1, Atg5, and Atg7 were downregulated in *Blmh*^-/-^ and *Blmh*^-/-^5xFAD brains. Aβ was elevated in *Blmh*^-/-^5xFAD brains. These biochemical changes were recapitulated in *Blmh*-silenced N2a-APP_swe_ cells, which also showed increased H4K20me1-*mTOR* promoter binding and impaired autophagy flux (Lc3-I, Lc3-II, p62). *Phf8*-silencing or treatments with Hcy-thiolactone or *N*-Hcy-protein, metabolites elevated in *Blmh*^-/-^ mice, induced biochemical changes in N2a-APP_swe_ cells like those induced by the *Blmh*-silencing. However, *Phf8*-silencing elevated Aβ without affecting AβPP.

**Conclusions:**

Our findings show that Blmh interacts with AβPP and the Phf8/H4K20me1/mTOR/autophagy pathway, and that disruption of those interactions causes Aβ accumulation and cognitive/neuromotor deficits.

## INTRODUCTION

Bleomycin hydrolase (Blmh), named for its ability to deaminate and inactivate the anticancer glycopeptide drug bleomycin, is a thiol-dependent cytoplasmic aminopeptidase expressed in human and rodent organs, including the brain [1, 2]. In addition to the aminopeptidase activity, Blmh has a thiolactonase activity and takes part in homocysteine (Hcy) metabolism by detoxifying Hcy-thiolactone [3, 4].

Hcy-thiolactone is formed from Hcy in an error-editing reaction in protein biosynthesis catalyzed by methionyl-tRNA synthetase [5–7]. Accumulation of Hcy-thiolactone is harmful because of its ability to modify protein lysine residues [8], which generates structurally- and functionally-impaired *N*-homocysteinylated (*N*-Hcy)-proteins with proinflammatory, prothrombotic, and pro-amyloidogenic properties [6]. Hcy-thiolactone and *N*-Hcy-proteins accumulate in intellectually disabled cystathionine β-synthase- and methylenetetrahydrofolate reductase-deficient patients [6] and are mechanistically linked to neurological diseases such as Alzheimer’s disease (AD) [9, 10], stroke [11], cognitive impairment [12], Parkinson’s disease [13], and neural tube defects [14, 15], as well as cardiovascular disease [16], cancer [17–19], and rheumatoid arthritis [20].

BLMH has been linked to AD and Huntington’s disease. Specifically, BLMH has the ability to process amyloid-β protein precursor (AβPP) to amyloid-β (Aβ) [21] and to further process Aβ [22]. BLMH has also the ability to generate *N*-terminal fragments of huntingtin, thought to be important mediators of the pathogenesis of Huntington’s disease [23]. In the human brain, BLMH is localized in neocortical neurons and in dystrophic neurites of senile plaques [24]. A single nucleotide polymorphism in human *BLMH* gene, resulting in I443 V substitution in the BLMH protein, is associated with an increased risk of AD [25, 26]; however, no association was reported in other studies [27–29].

The Hcy-thiolactonase and aminopeptidase activities of BLMH are decreased in brains of AD patients, suggesting that the attenuated BLMH activity could contribute to the pathology of AD [30]. In mice, deletion of the *Blmh* gene causes astrogliosis and behavioral changes [31]. Furthermore, *Blmh*^-/-^ mice exhibit diminished ability to detoxify Hcy-thiolactone, which elevates brain Hcy-thiolactone levels, and increases neurotoxicity of Hcy-thiolactone injections [4]. Studies of *Blmh*^-/-^ mouse brain proteome demonstrated that Blmh interacts with diverse cellular processes, such as synaptic plasticity, cytoskeleton dynamics, cell cycle, energy metabolism, and antioxidant defenses that are essential for brain homeostasis [9]. Collectively, these findings suggest that Blmh plays a key role in the central nervous system (CNS).

Plant homeodomain finger protein 8 (PHF8) has been identified as one of the X chromosome genes linked to intellectual disability syndrome, autism spectrum disorder, attention deficit hyperactivity disorder [32], and severe mental retardation [33]. PHF8 is a histone demethylase that can demethylate H4K20me1, H3K9me2/me1, and H3K27me2. Demethylation of H4K20me1 by PHF8 is important for supporting homeostasis of mTOR signaling. The phenotype of human PHF8 deficiency has been replicated in *Phf8*^-/-^ mice, which show impaired hippocampal long-term potentiation and behavioral deficits in learning and memory [34].

In the present work we examined the role of Blmh in the CNS by studying behavioral and biochemical consequences of *Blmh* gene deletion in mice. Since dysregulated mTOR signaling and autophagy have been implicated in Aβ accumulation in AD brains [35–38], and H4K20me1 demethylation by PHF8 is important for maintaining homeostasis of mTOR signaling, we examined how these processes are affected in brains of *Blmh*^-/-^ *versus Blmh^+^*^/+^ mice as well as transgenic *Blmh*^-/-^5xFAD *versus Blmh^+^*^/+^5xFAD mice. We also examined how biochemical changes in these processes and in APP/Aβ expression relate to behavioral performance in *Blmh*^-/-^5xFAD mice. We studied underlying molecular mechanisms by manipulating Blmh or Phf8 expression or Hcy-thiolactone and *N*-Hcy-protein levels in mouse neuroblastoma N2a-APPswe cells.

## MATERIALS AND METHODS

### Mice and treatments

*Blmh*^-/-^ [39] and 5xFAD [40] mice on the C57BL/6J genetic background were housed and bred at the Rutgers-New Jersey Medical School Animal Facility. 5xFAD mice overexpress the K670 N/M671 L (Swedish), I716 V (Florida), and V717I (London) mutations in human APP(695), and M146 L and L286 V mutations in human PS1 associated with familial early-onset AD. 5xFAD mice accumulate elevated levels of Aβ_42_ beginning around 2 months of age [40] (https://www.alzforum.org/research-models/5xfad-b6sjl). The *Blmh*^-/-^ mice were crossed with 5xFAD animals and the resulting heterozygotes were used to generate *Blmh*^-/-^5xFAD mice and their *Blmh^+^*^/+^5xFAD sibling controls, hemizygous for the 5xFAD transgene. Mouse *Blmh* genotype were established by PCR of tail clips using the following primers: *Blmh* intron 2 forward primer p1 (5^′^-CACTGTAGCTGTACTCACAC), *Blmh* exon 3 reverse primer p2 (5^′^-GCGACAGAGTACCATGTAGG-3^′^) and neomycin cassette reverse primer p3 (5^′^-ATTTGTCACGTCCTGCACGACG-3^′^) [39]. 5xFAD genotype was proven using human AβPP and PS1 primers (hAPP forward 5^′^-AGAGTACCAACTTGCATGACTACG-3^′^ and reverse 5^′^-ATGCTGGATAACTGCCTTCTTATC-3^′^; hPS1 forward 5^′^-*GCTTTTTCCAGCTCTCATTTACTC*-3^′^ *and reverse* 5^′^-*AAAATTGATGGAATGCTAATT GGT*-3^′^). The mice were fed with a standard rodent chow (LabDiet5010; Purina Mills International, St. Louis MO, USA) [4]. Two- and four-month-old *Blmh*^-/-^ mice and their *Blmh*^+/+^ siblings, as well as 5- and 12-month-old *Blmh*^-/-^5xFAD mice and their *Blmh^+^*^/+^5xFAD siblings were used in experiments. Hyperhomocysteinemia (HHcy) was induced pharmacologically, as needed, by providing drinking water supplemented with 1% methionine (a ‘high Met diet’) [41–43] to mice starting at 1 month of age. The high Met diet significantly increases plasma total Hcy levels (*p* < 1.E-06) (6-fold from 6.8 to 39μM in *Blmh*^-/-^ mice and 10-fold from 7.4 to 77μM in *Blmh*^+/+^ mice) as well as *N*-Hcy-protein levels (*p* < 0.001) (3-fold from 2.8 to 8.4μM in *Blmh*^-/-^ mice and 4.5-fold from 1.2 to 5.4μM in *Blmh*^+/+^ mice) [4]. The groups were derived from multiple litters and equal number of males and females were used in each group. Animal procedures were approved by the Institutional Animal Care and Use Committee at Rutgers-New Jersey Medical School.

### Behavioral testing

*Novel Object Recognition test.* NOR is a test of recognition memory [44]. The test was conducted in two sessions, divided by a 6-h intersession interval. During the first session (familiarization session), the animal was free to explore two similar objects, and during the second session (test session), one of the objects was replaced by a novel, unfamiliar object. No habituation phase was performed. A minimal exploration time for both objects during both the familiarization and test phase (∼20 s) was used, with a maximal time of 10 min to reach the criterion. Mice were evaluated in a white plastic box (33×33×20 cm). W used objects that differ in shape and texture: towers of Lego bricks (8-cm high and 3.2-cm wide, built-in blue, yellow, red, and green bricks) and Falcon tissue culture flasks filled with sand (9.5 cm high, 2.5 cm deep and 5.5 cm wide, transparent plastic with a yellow bottle cap). We scored object exploration whenever the mouse sniffed the object or touched the object while looking at it (i.e., when the distance between the nose and the object was less than 2 cm). Climbing onto the object (unless the mouse sniffs the object it has climbed on) or chewing the object did not qualify as exploration.

*Hindlimb test.* The hindlimb clasping test is used to assess neurodegeneration in mouse models [45]. For this test, mice were suspended by the base of the tail and videotaped for 10 s. Three separate trials were taken over three consecutive days. Hindlimb clasping was scored from 0 to 3 : 0 = hindlimbs splayed outward and away from the abdomen, 1 = one hindlimb retracted inwards towards the abdomen for at least 50% of the observation period, 2 = both hindlimbs partially retracted inwards towards the abdomen for at least 50% of the observation period, 3 = both hindlimbs completely retracted inwards towards the abdomen for at least 50% of the observation period. Hindlimb clasping scores were added together for the three separate trials.

*Ledge test.* The ledge test is used to assess motor deficits in rodent models of CNS disorders [46]. Typically, mice walk along the ledge of a cage and try to descend back into the cage. Three separate trials were taken for each mouse. Ledge test was scored from 0 to 3 points: 0 = a mouse walked along the ledge without slipping and lowered itself back into the cage using paws; 1 = the mouse lost its footing during walking along the ledge but otherwise appeared coordinated; 2 = the mouse did not effectively use its hind legs and landed on its head rather than paws when descending into the cage; 3 = the mouse fell of the ledge or was shaking, barely moving.

*Cylinder test.* The cylinder test is used to assess sensorimotor function in rodent models of CNS disorders. A mouse was placed in the transparent 500 ml plastic cylinder. The number of times the mouse reared up and touched the cylinder wall during a period of 3 min was counted. A rear is defined as a vertical movement with both forelimbs off the floor so that the mouse is standing only on its hindlimbs. At the end of 3 min, the mouse was removed and placed back into its home cage. Because spontaneous activity in the cylinder is affected by repeated testing resulting in reduced activity over time, mice were only once in their lifetime.

### Brain protein extraction

Mice were euthanized by CO_2_ inhalation, the brains collected and frozen on dry ice. Frozen brains were pulverized with dry ice using a mortar and pestle and stored at –80°C. Proteins were extracted from the pulverized brains (50±5 mg; 30±3 mg brain was used for Aβ analyses) using RIPA buffer (4 v/w, containing protease and phosphatase inhibitors) with sonication (Bandelin SONOPLUS HD 2070) on wet ice (three sets of five 1-s strokes with 1 min cooling interval between strokes). Brain extracts were clarified by centrifugation (15,000 g, 30 min, 4°C) and clear supernatants having 8-12 mg protein/mL were collected (RIPA-soluble fraction). Protein concentrations were measured with BCA kit (Thermo Scientific).

For Aβ analyses, pellets after protein extraction with RIPA buffer were re-extracted by brief sonication in 2% SDS, centrifuged (15,000 g, 15 min, room temperature (RT)), and the supernatants again collected (SDS-soluble fraction). The SDS-extracted pellets were then extracted by sonication in 70% formic acid (FA), centrifuged, and the supernatants were collected (the FA-soluble fraction) [47].

### Cell culture and treatments

Mouse neuroblastoma N2a-APPswe cells, harboring a human AβPP transgene with the K670 N and M671 L Swedish mutations associated with familial early-onset AD [48] were grown (37°C, 5% CO_2_) in DMEM/F12 medium (Thermo Scientific) supplemented with 5% fetal bovine serum, non-essential amino acids, and antibiotics (penicillin/streptomycin) (MilliporeSigma).

After cells reached 70-80% confluency, the monolayers were washed twice with PBS and overlaid with DMEM medium without methionine (Thermo Scientific), supplemented with 5% dialyzed fetal bovine serum (MilliporeSigma) and non-essential amino acids. L-Hcy-thiolactone (MilliporeSigma) or *N*-Hcy-protein, prepared as described in [49], were added (at concentrations indicated in figure legends) and the cultures were incubated at 37°C in 5% CO_2_ atmosphere for 24 h.

For gene silencing, *Blmh*-targeting siRNAs (Cat. # 100821 and s63474) or *Phf8* gene (Cat. # S115808, and S115809) (Thermo Scientific) were transfected into cells kept in Opti-MEM medium by 24-h treatments with Lipofectamine RNAiMax (Thermo Scientific). Cellular RNA for RT-qPCR analyses were isolated as described in section 2.5 below. For protein extraction, RIPA buffer (MilliporeSigma) was used according to manufacturer’s protocol.

### Western blots

Proteins were separated by SDS-PAGE on 10% gels (20μg protein/lane) and transferred to PVDF membrane (0.2μm; Bio-Rad, cat. # 1620177) for 20 min at 0.1 A, 25 V using Trans Blot Turbo Transfer System (Bio-Rad). After blocking with 5% bovine serum albumin in 1X Tris-Buffered Saline, 0.1% Tween 20 Detergent buffer (TBST; 1 h, RT), the membranes were incubated overnight at 4°C with anti-Blmh (Abcam, AB188371), anti-Phf8 (Abcam, ab36068), anti-H4K20me1 (Abcam ab177188), anti-mTOR (CS #2983), anti-pmTOR Ser2448 (CS, #5536), anti-Atg5 (CS, #12994), anti-Atg7 (CS, #8558), anti-Bcln1 (CS, #3495), anti-Lc3 (CS, #4599) anti-p62 (CS, #23214), anti-Gapdh (CS, #5174), or anti-Aβpp (Abcam, ab126732) for 1 h. Membranes were washed three times with TBST, 10 min each, and incubated with goat anti-rabbit IgG secondary antibody conjugated with horseradish peroxidase. Positive signals were detected using Western Bright Quantum-Advansta K12042-D20 and GeneGnome XRQ NPC chemiluminescence detection system. Bands intensity was calculated using Gene Tools program from Syngene.

For western blots analyses of Aβ, brain protein extracts (2μL) were separated on 10% Tricine gels, and then transferred (0.5 A, 25 V 10 min) onto 22μm PVDF membranes (Bio-Rad). The membranes were washed 3 times with 1x TBST and then blocked with 5% bovine serum albumin for 1 h at RT. After blocking, membranes were washed 3 times with 1x TBST and then incubated with primary anti-Aβ antibody (D54D2, CS #8243). Membranes were washed 3 times with 1x TBS-T and incubated with anti-rabbit IgG HRP-linked antibodies (CS#7074) for 1 h at RT. Signals were collected using clarity Max Western ECL Substrate (Bio-Rad) and GeneGnome XRQ - Chemiluminescence imaging (Syngene).

### RNA isolation, cDNA synthesis, RT-qPCR analysis

Total RNA was isolated using Trizol reagent (MilliporeSigma). cDNA synthesis was conducted using Revert Aid First cDNA Synthesis Kit (Thermo Fisher Scientific) according to manufacturer’s protocol. Nucleic acid concentration was measured using NanoDrop (Thermo Fisher Scientific). RT-qPCR was performed with SYBR Green Mix and CFX96 thermocycler (Bio-Rad). The 2^(- ΔΔCt)^ method was used to calculate the relative expression levels [50]. Data analysis was performed with the CFX Manager™ Software, Microsoft Excel, and GraphPad Prism7. RT-qPCR primer sequences are listed in Supplementary Table 1.

### Chromatin immunoprecipitation assay

For the CHIP assays, CUT&RUN Assay Kit #86652 (Cell Signaling Technology, Danvers, MA, USA) was used following the manufacturer’s protocol. Each assay was done in triplicates. cells. Cells (100 000/ assay) were trypsinized, harvested, washed 3x in ice-cold PBS, and bound to concanavalin A-coated magnetic beads for 5 min, RT. Cells were then incubated (4 h, 4°C) with 2.5μg of anti-PHF8 antibody (Abcam, ab36068) or anti-H4K20me1 antibody (Abcam, ab177188) in the antibody-binding buffer plus digitonin that permeabilizes cells. Next, cells are treated with pAG-MNase (1 h, 4°C), washed, and treated with CaCl2 to activate DNA digestion (0.5 h, 4°C). Cells were then treated with the stop buffer and spike-in DNA was added for each reaction for signal normalization, and incubated (30 min, 37°C). Released DNA fragments were purified using DNA Purification Buffers and Spin Columns (CS #14209) and quantified by RT-qPCR using primers targeting the promoter, upstream, and downstream regions of the *mTOR* gene (Supplementary Table 1).

### Confocal microscopy, Aβ staining in N2a-APPswe cells

Mouse neuroblastoma N2a-APPswe cells were cultured in Millicell EZ SLIDE 8-well glass slides (Merck). After 24 h treatments, cells were washed with PBS (3 times, 10 min each) and fixed with 4% PFA (Sigma-Aldrich) (37°C, 15 min). After fixation, cells were again washed 3 times with PBS buffer and permeabilized in 0.1% Triton X-100 solution (RT, 20 min), blocked with 0.1% bovine serum albumin (RT, 1 h), and incubated with anti-Aβ antibody (CS #8243; 4°C, 16 h). Cells were then washed 3 times with PBS and stained with secondary antibody Goat Anti-Rabbit IgG H&L (Alexa Fluor® 488) (Abcam, ab150077; RT, 1 h) to detect Aβ. DAPI (Vector Laboratories) was used to visualize nuclei. Fluorescence signals were detected by using a Zeiss LSM 880 confocal microscope with a 488 nm filter for the Alexa Fluor® 488 (Aβ) and 420–480 nm filter for DAPI, taking a *z* stack of 20-30 sections with an interval of 0.66μm and a range of 15μm. Zeiss Plan-Apochromat X40/1.2 Oil differential interference contrast objective were used for imaging. Images were quantified with the ImageJ Fiji software (NIH). Negative controls without primary or secondary antibody, which yielded no fluorescent signals, and positive controls with an authentic Aβ_42_ standard (1μg/mL medium) (Abcam, ab120301), which showed fluorescent signals, were done to verify the specificity of the assay.

### Statistical analysis

Data *from in vivo* and *in vitro* experiments are reported as mean ± standard deviation (SD). Values for each experimental/treatment group were normalized to controls. Data were analyzed using one-way analysis of variance (ANOVA) with Tukey’s multiple comparisons post-test using GraphPad Prism7 software (GraphPad Holdings LLC, San Diego CA, USA, https://www.graphpad.com). A two-sided unpaired Student *t* test was used for analysis of NOR data.

## RESULTS

### Blmh gene deletion impairs recognition memory, induces neurodegeneration, and sensorimotor deficits in Blmh^-/-^ and Blmh^-/-^5xFAD mice

**Fig. 1:**
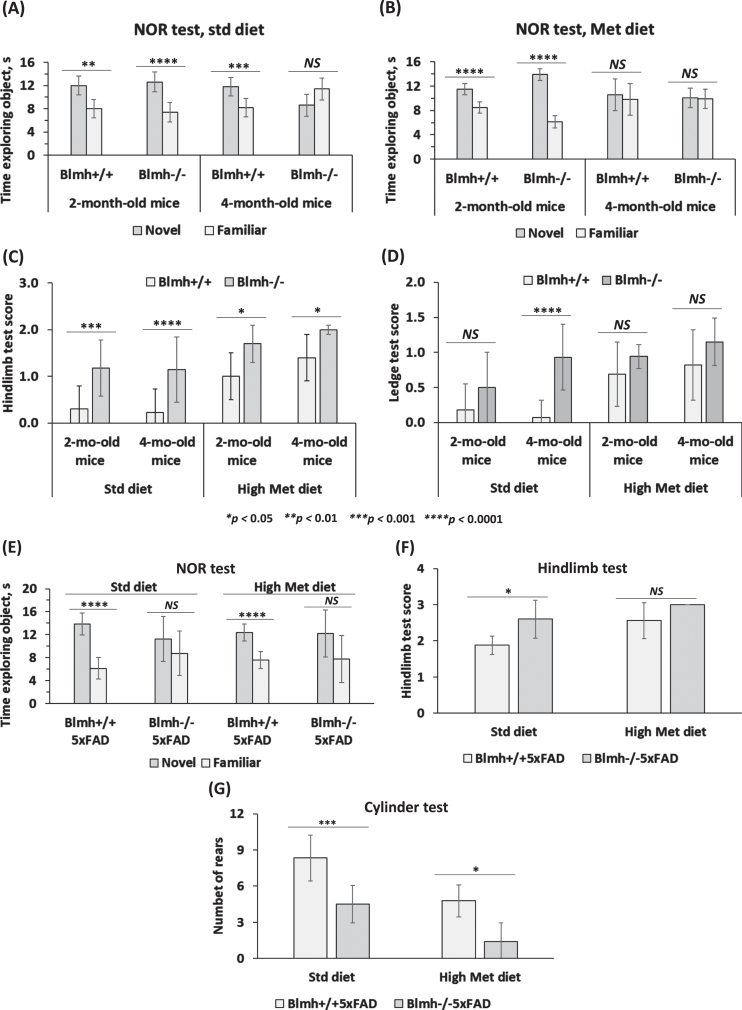
Deletion of *Blmh* gene impairs recognition memory and sensorimotor activity. A-D) *Blmh*^-/-^ *versus Blmh*^+/+^ mice. A, B) Novel object recognition (NOR) test: Time spent at novel and familiar objects. Std diet: *N* = 6, 7, 9, and 4 mice/group. Met diet: 7, 7, 8, and 10 mice/group. C) Hindlimb clasping test scores. D) Ledge test scores. Std diet: *N* = 8, 9, 30, and 14 mice/group. Met diet: 20, 11, 14, and 10 mice/group. E-G) *Blmh*^-/-^5xFAD *versus Blmh*^+/+^5xFAD mice - (E) NOR test: Time spent exploring novel and familiar objects. Std diet: *N* = 8 and 4 mice/group; Met diet: 8 and 5 mice/group. F) Hindlimb clasping test scores. Std diet: *N* = 8 and 4; Met diet: 9 and 7 mice/group. G) Cylinder test: number of rears. Std diet: *N* = 8 and 6; Met diet: 9 and 5 mice/group. *p*-values for the NOR test were calculated by the paired two-sided Student t test. *p*-values for the hindlimb, ledge, and cylinder tests were calculated by one-way ANOVA with Tukey’s multiple comparisons test. *p<0.05, **p<0.01, ***p<0.001, or ****p<0.0001.

*Blmh**^-/-^** mice.* To examine effects of Blmh depletion on cognition and sensorimotor activity, *Blmh*^-/-^ and *Blmh*^+/+^ mice fed with a control or high Met diet were assessed in the NOR, hindlimb clasping, and ledge tests. We found that 4-month-old *Blmh*^-/-^ mice did not differentiate between novel and familiar objects in the NOR test, regardless of the diet (Fig. 1A, B), showing impaired recognition memory. As expected, 4-month-old control *Blmh*^+/+^ mice showed normal preference for novelty (Fig. 1A); however, the preference for novelty disappeared when the mice were fed with a high Met diet (Fig. 1B). In contrast, 2-month-old *Blmh*^-/-^ mice showed significant preference for a novel object, regardless of the diet, as did 2-month-old *Blmh*^+/+^ mice (Fig. 1A, B). These findings show that a longer exposure (>2-month) is needed for the manifestation of detrimental effects of Blmh depletion or high Met diet on cognition.

The hindlimb test showed more severe clasping (significantly higher scores) in *Blmh*^-/-^ mice *versus* their *Blmh*^+/+^ siblings (Fig. 1C), showing neurological impairment in *Blmh*^-/-^ animals. High Met diet significantly increased the hindlimb scores, with greater increases in *Blmh*^+/+^ than in *Blmh*^-/-^ mice, resulting in attenuated *Blmh*^-/-^ versus *Blmh*^+/+^ difference in high Met diet animals (Fig. 1C). Overall, high Met diet attenuated effects of the *Blmh*^-/-^ genotype on the hindlimb score.

The ledge test showed significantly higher scores for 4-month-old, but not in 2-month-old, *Blmh*^-/-^ versus *Blmh*^+/+^ mice (Fig. 1D), indicating neuromotor deficiency in *Blmh*^-/-^ animals. High Met diet increased the ledge test scores, with greater increases in *Blmh*^+/+^ than in *Blmh*^-/-^ mice, resulting in attenuated *Blmh*^-/-^ versus *Blmh*^+/+^ difference in high Met diet animals (Fig. 1D). Overall, high Met diet attenuated effects of the *Blmh*^-/-^ genotype on the ledge test score.

*Blmh^-/-^5xFAD mice.* We also examined effects of Blmh depletion on cognition and neuromotor activity in 1-year-old *Blmh*^-/-^5xFAD *versus Blmh*^+/+^5xFAD mice. We found that *Blmh*^-/-^5xFAD mice fed with a standard chow or high Met diet did not differentiate between novel and familiar objects in the NOR test (Fig. 1E), showing impaired recognition memory in *Blmh*^-/-^5xFAD animals. In contrast, *Blmh*^+/+^5xFAD mice fed with a standard chow diet or high Met diet showed normal preference for novelty (Fig. 1E).

The hindlimb clasping test showed significantly higher scores in *Blmh*^-/-^5xFAD *versus Blmh*^+/+^5xFAD mice fed with a standard chow or high Met diet (Fig. 1F), showing that *Blmh*^-/-^ genotype promotes neuromotor deficits in *Blmh*^-/-^5xFAD animals. High Met diet increased the hindlimb scores, with greater increase in *Blmh*^+/+^5xFAD than in *Blmh*^-/-^5xFAD mice and abrogated the *Blmh*^-/-^5xFAD versus *Blmh*^+/+^5xFAD difference (Fig. 1F).

The cylinder test showed significantly reduced scores for *Blmh*^-/-^5xFAD *versus Blmh*^+/+^5xFAD mice fed with a standard chow or high Met diet (Fig. 1G), showing that *Blmh*^-/-^ genotype promotes neuromotor deficits in *Blmh*^-/-^5xFAD animals. High Met diet reduced the cylinder test scores, with greater relative reductions in *Blmh*^-/-^5xFAD mice, resulting in increased *Blmh*^-/-^5xFAD *versus Blmh*^+/+^5xFAD difference in high Met diet animals (Fig. 1G).

### Blmh depletion downregulates histone demethylase Phf8 and upregulates H4K20me1 epigenetic mark in brains of Blmh^-/-^ and Blmh^-/-^5xFAD mice

**Fig. 2:**
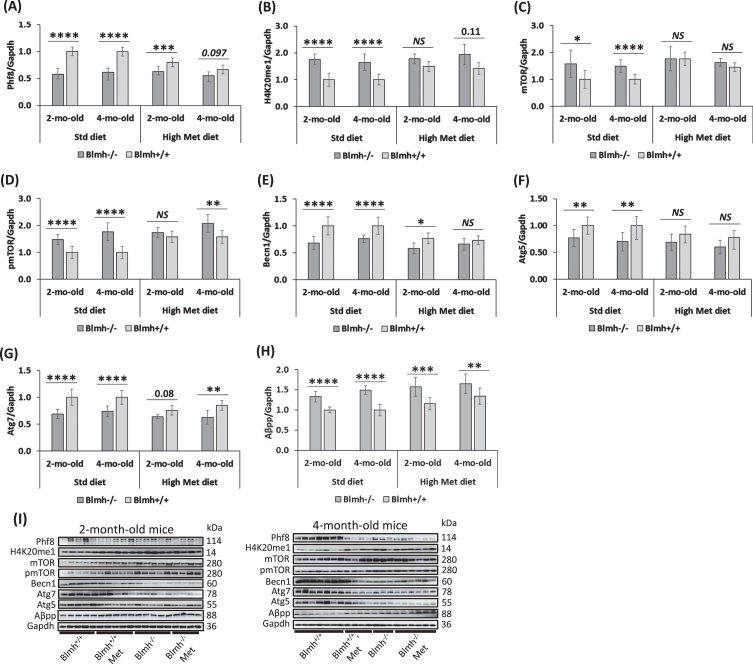
Blmh depletion affects the expression of histone demethylase Phf8, histone H4K20me1 epigenetic mark, mTOR signaling, autophagy, and Aβpp in the *Blmh*^-/-^ mouse brain. One-month-old *Blmh*^-/-^ mice (*n* = 7 and 14) and *Blmh*^+/+^ sibling controls (*n* = 10 and 10) fed with a control or high Met diet (to induce HHcy) for one or three months were used in experiments. Each group included 7-14 mice of both sexes. Bar graphs illustrating quantification of the following brain proteins by western blotting are shown: Phf8 (A), H4K20me1 (B), mTOR (C), pmTOR (D), Becn1 (E), Atg5 (F), Atg7 (G), and Aβpp (H). Gapdh protein was used as a reference for normalization. Representative pictures of western blots used for protein quantification are shown in panel (I). Each mouse was assayed in three independent experiments and average values were used to calculate the mean ± standard deviation (SD) values for wrote down proteins in each experimental group. *p<0.05, **p<0.01, ***p<0.001, or ****p<0.0001 were calculated by one-way ANOVA with Tukey’s multiple comparisons test. The numbers above bars show p values 0.08 –0.11. *NS*, not significant.

The present findings that Blmh-deleted mice showed behavioral deficits in memory that are like cognitive deficits in memory seen in Phf8-deleted mice [34] suggests that Blmh may interact with Phf8. To examine this possibility, we quantified Phf8 protein in brains of young *Blmh*^-/-^ mice and their *Blmh*^+/+^ sibling controls by western blotting. We also examined how HHcy, induced by supplying 1% methionine (Met) in drinking water, affects the Blmh-Phf8 interaction. We found that Phf8 protein was significantly downregulated in brains of *Blmh*^-/-^ mice *versus Blmh*^+/+^ sibling controls in mice fed with a standard chow diet (Fig. 2A). The reduced Phf8 expression in *Blmh*^-/-^ *versus Blmh*^+/+^ brains was attenuated in mice fed with a high Met diet (Fig. 2A).

However, Phf8 expression in *Blmh*^-/-^ mice was not affected by high Met diet (Fig. 2A). In contrast, in *Blmh*^+/+^ mice, high Met diet significantly downregulated Phf8 levels (Fig. 2A), showing that the Blmh-Phf8 interaction is affected by high Met diet.

The histone H4K20me1 epigenetic mark, which is controlled by Phf8 [34], was significantly upregulated in brains of *Blmh*^-/-^ *versus Blmh*^+/+^ mice fed with a standard chow diet (Fig. 2B). High Met diet significantly elevated H4K20me1 levels in *Blmh*^+/+^ mice but not in *Blmh*^-/-^ animals, thereby abrogating the effect of *Blmh*^-/-^ genotype on H4K20me1 (Fig. 2B).

To figure out how the Blmh-Phf8 interaction is affected by Aβ accumulation, we quantified Phf8 in brains of *Blmh*^-/-^ and *Blmh*^+/+^ mice on the 5xFAD background. We found significant downregulation of Phf8 (Supplementary Figure 1A) and upregulation of H4K20me1 (Supplementary Figure 1B) in *Blmh*^-/-^5xFAD *versus Blmh*^+/+^5xFAD mice, like those seen in *Blmh*^-/-^ *versus Blmh*^+/+^ animals (Fig. 1A, B). High Met diet abrogated effects of *Blmh* deletion on Phf8 and H4K20me1 levels in 12-month-old but not in 5-month-old *Blmh*^-/-^5xFAD mice (Supplementary Figure 1A, B). These findings show that the Blmh-Phf8 interaction is independent of Aβ accumulation but is influenced by high-Met-diet.

### Blmh deletion upregulates mTOR signaling and inhibits autophagy in brains of Blmh^-/-^ and Blmh^-/-^5xFAD mice

*Blmh^-/-^ mice.* Because Phf8/H4K20me1 regulate mTOR signaling [34], we next examined effects of *Blmh* deletion on levels of mTOR and its active form, phosphorylated at Ser2448 (pmTOR). We found that mTOR protein was significantly upregulated in brains of *Blmh*^-/-^ *versus Blmh*^+/+^ mice (Fig. 2C). The effect of *Blmh*^-/-^ genotype on mTOR expression was abrogated by high Met diet (Fig. 2C).

High Met diet significantly increased mTOR expression in *Blmh*^+/+^ mice (Fig. 2C). However, high Met diet did not affect mTOR levels in *Blmh*^-/-^ animals (Fig. 2C).

pmTOR was also significantly elevated in brains of *Blmh*^-/-^ *versus Blmh*^+/+^ mice (Fig. 2D). The effect of *Blmh*^-/-^ genotype on pmTOR levels was abrogated by high Met diet in 2-month-old but not in 4-month-old mice.

High Met diet significantly elevated pmTOR levels in 2- and 4-month-old *Blmh*^+/+^ mice and in 2-month-old *Blmh*^-/-^ animals (Fig. 2D). These findings show that the elevation of pmTOR levels reflects the upregulation of mTOR in *Blmh*^-/-^ mice.

Because mTOR is a major regulator of autophagy, we quantified effects of Blmh depletion on autophagy-related proteins. We found that the regulators of autophagosome assembly Becn1, Atg5, and Atg7 were significantly downregulated in brains of *Blmh*^-/-^ *versus Blmh*^+/+^ mice (Fig. 2E, F, G). The effects of *Blmh*^-/-^ genotype on autophagy were attenuated (Becn1, Atg7) or abrogated (Atg5) by high Met diet.

*Blmh^-/-^5xFAD mice.* We found that mTOR and pmTOR were significantly upregulated also in brains of *Blmh*^-/-^5xFAD versus *Blmh*^+/+^5xFAD mice (Supplementary Figure 1C, D). High Met diet abrogated these effects on pmTOR in young (5-month-old) and old (12-month-old) mice (Supplementary Figure 1D), and on mTOR in old (12-month-old) *Blmh*^-/-^5xFAD animals (Supplementary Figure 1C). High Met diet did not affect mTOR and pmTOR levels in *Blmh*^-/-^5xFAD mice. However, high Met diet upregulated mTOR (Supplementary Figure 1C) and pmTOR (Supplementary Figure 1D) in 12-month-old, but not in 5-month-old, *Blmh*^+/+^5xFAD mice.

Markers of autophagy such as Beclin1, Atg5, and Atg7 were downregulated in 5- and 12-month-old *Blmh*^-/-^5xFAD versus *Blmh*^+/+^5xFAD mice (Supplementary Figure 1E-G), like in young *Blmh*^-/-^ versus *Blmh*^+/+^ animals (Fig. 2E-G). Protein p62, a receptor for degradation of ubiquitinated substrates, was upregulated in 5-month-old, and to a lesser extent in 12-month-old *Blmh*^-/-^5xFAD mice (Supplementary Figure 1H).

Becn1 and Atg5 (Supplementary Figure 1E, F) were also downregulated in *Blmh*^-/-^5xFAD versus *Blmh*^+/+^5xFAD mice fed with high Met diet. However, high Met diet abrogated effects of the *Blmh*^-/-^ genotype on Atg7 (in 5- and 12-month-old mice, Supplementary Figure 1G) and p62 (in 12-month-old mice, Supplementary Figure 1H) in 5xFAD mice.

### Blmh gene deletion upregulates Aβpp in Blmh^-/-^ and AβPP in Blmh^-/-^5xFAD mouse brain

We found that Aβpp was significantly upregulated in brains of *Blmh*^-/-^ *versus Blmh*^+/+^ mice fed with a standard chow diet (Fig. 2H). Aβpp was similarly upregulated in *Blmh*^-/-^ *versus Blmh*^+/+^ mice fed with high Met diet (Fig. 2H).

We saw a similarly upregulated AβPP in 5- and 12-month-old *Blmh*^-/-^5xFAD mice carrying a mutated human AβPP transgene (Supplementary Figure 1I). High Met diet had no effect on AβPP in brains of 5-month-old *Blmh*^-/-^5xFAD and *Blmh*^+/+^5xFAD mice. However, AβPP was upregulated in brains of 12-month-old *Blmh*^+/+^5xFAD mice by high Met diet, which abrogated the difference in AβPP between *Blmh*^-/-^5xFAD versus *Blmh*^+/+^5xFAD animals seen in animals fed with a control diet (Supplementary Figure 1I).

### Blmh controls the expression of mTOR-, autophagy-related proteins, and AβPP in N2a-APPswe cells

To elucidate the mechanism by which Blmh depletion impacts Phf8 and its downstream effects on mTOR, autophagy, and AβPP, we first examined whether our findings in *Blmh*^-/-^ mice can be recapitulated in cultured mouse neuroblastoma N2a-APPswe cells carrying a mutated human AβPP transgene. We silenced the *Blmh* gene in these cells by RNA interference using two different siRNA (siRNA Blmh#1 and siRNA Blmh#2) for transfections; controls were mock-transfected or transfected with scrambled siRNA (siRNAscr). The changes in gene expression at the protein and mRNA levels in *Blmh*-silenced versus control cells were analyzed by western blotting and RT-qPCR using Gapdh protein and mRNA, respectively, as a reference.

We found that the Blmh protein level was significantly reduced in *Blmh*-silenced cells (by 80%; Supplementary Figure 2A). We also found that the histone demethylase Phf8 protein level was also significantly reduced (Supplementary Figure 2B), while the histone H4K20me1 level was significantly elevated (Supplementary Figure 2C) in *Blmh*-silenced cells.

At the same time, mTOR protein was significantly upregulated in *Blmh*-silenced cells (Supplementary Figure 2D), as were pmTOR (Supplementary Figure 2E) and AβPP (Supplementary Figure 2F). Autophagy-related proteins Becn1, Atg5, and Atg7 were significantly downregulated in *Blmh*-silenced cells (Supplementary Figure 2G, H, I).

To find whether Blmh depletion affects the autophagy flux, we quantified Lc3 and p62 in *Blmh*-silenced N2a-APPswe cells. We found significant reductions in soluble Lc3-I (Supplementary Figure 2J) and an autophagosome-bound lipidated Lc3-II (Supplementary Figure 2K) in *Blmh*-silencing cells. The Lc3-II/Lc3-I ratio was also significantly reduced (Supplementary Figure 2L), while p62 was upregulated (Supplementary Figure 2M). These findings show that Blmh depletion impairs the autophagy flux.

To elucidate whether Blmh exerts a transcriptional control over the expression of AβPP, mTOR- and autophagy-related proteins, we quantified by RT-qPCR mRNAs for these proteins. We found that the changes in mRNA levels in *Blmh*-silenced N2a-APPswe cells (Supplementary Figure 3) were like the changes in the corresponding protein levels (Supplementary Figure 2). Specifically, Blmh mRNA was significantly reduced (by 95%; Supplementary Figure 3A), as was Phf8 mRNA (Supplementary Figure 3B). mTOR mRNA was significantly upregulated (Supplementary Figure 3C) as was AβPP mRNA (Supplementary Figure 3D) in *Blmh*-silenced cells, reflecting changes in the corresponding protein levels in these cells (Supplementary Figure 2A-F). mRNAs for autophagy-related proteins Atg5, Atg7, and Becn1 (Supplementary Figure 3E, F, and G, respectively).

These findings show that Blmh transcriptionally controls the expression of these proteins and prove that the changes in Phf8, H4K20m1, mTOR signaling, autophagy, and AβPP induced by *Blmh* gene silencing in N2a-APPswe cells (Supplementary Figure 2) recapitulate the *in vivo* findings in *Blmh*^-/-^ (Fig. 2) *Blmh*^-/-^ 5xFAD Supplementary Figure 1) brains.

### Hcy-thiolactone and N-Hcy-protein modulate the expression of AβPP, mTOR- and autophagy-related proteins in N2a-APPswe cells

The effects of *Blmh* deletion on Phf8 and its downstream targets can be caused by the absence the Blmh protein or by Hcy-thiolactone and *N*-Hcy-protein, which are known to be elevated in *Blmh*^-/-^ mice [4]. To distinguish between these possibilities, we treated N2a-APPswe cells with different concentrations of Hcy-thiolactone or *N*-Hcy-protein.

**Fig. 3:**
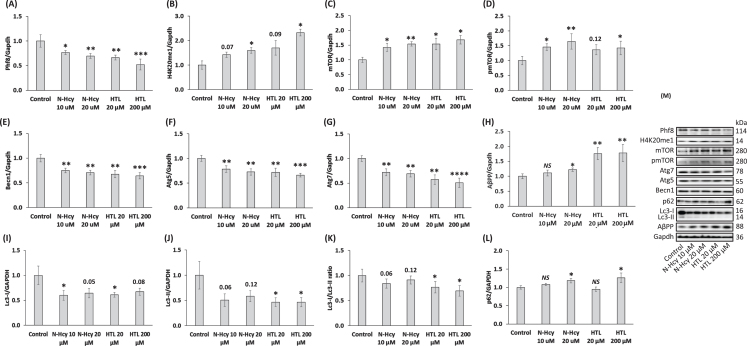
Hcy-thiolactone and *N*-Hcy-protein downregulate Phf8, upregulate the H4K20me1 epigenetic mark, mTOR signaling, AβPP, and impair autophagy in mouse neuroblastoma N2a-APPswe cells. N2a-APPswe cells were treated with indicated concentrations of *N*-Hcy-protein (N-Hcy) or Hcy-thiolactone (HTL) for 24 h at 37°C. Bar graphs illustrating the quantification of Phf8 (A), H4K20me1 (B), mTOR (C), pmTOR (D), Becn1 (E), Atg5 (F), Atg7 (G), AβPP (H), Lc3-I (I), Lc3-II (J), Lc3-I/Lc3-II ratio (K), and p62 (L) based on western blot analyses are shown. Gapdh was used as a reference protein. Each assay was repeated three times in three independent experiments. Mean ± SD values for each treatment group are shown. *p*-values were calculated by one-way ANOVA with Tukey’s multiple comparisons test. *p<0.05, **p<0.01, ***p<0.001, or ****p<0.0001. The numbers above bars show *p* values 0.05 –0.16. *NS*, not significant.

We found significantly reduced Phf8 levels in N2a-APPswe cells treated with Hcy-thiolactone or *N*-Hcy-protein compared to untreated control cells (Fig. 3A). Levels of methylated histone H4K20me1 were elevated in Hcy-thiolactone or *N*-Hcy-protein treated cells compared to untreated controls (Fig. 3B).

mTOR levels were significantly upregulated in N2a-APPswe cells by treatments with Hcy-thiolactone or *N*-Hcy-protein, compared to untreated cells (Fig. 3C). Levels of pmTOR were also significantly upregulated by these treatments (Fig. 3D). Autophagy-related proteins Becn1 (Fig. 3E) Atg5 (Fig. 3F), and Atg7 (Fig. 3G) were significantly downregulated in cells treated with Hcy-thiolactone or *N*-Hcy-protein.

AβPP levels were significantly upregulated in N2a-APPswe cells treated with Hcy-thiolactone or *N*-Hcy-protein compared to untreated controls (Fig. 3H).

To find whether Hcy-thiolactone or *N*-Hcy-protein can affect the autophagy flux, we quantified Lc3 and p62 in N2a-APPswe cells treated with these metabolites. We found that treatments with Hcy-thiolactone or *N*-Hcy-protein reduced levels of soluble Lc3-I (Fig. 3I) and an autophagosome membrane-bound lipidated Lc3-II (Fig. 3J). The Lc3-II/Lc3-I ratio, a measure of autophagy flux, was also reduced (Fig. 3K), while p62 was upregulated (Fig. 3L). Representative images of western blots are shown in Fig. 3M.

Taken together, these findings suggest that Hcy-thiolactone and *N*-Hcy-protein, metabolites that are elevated in *Blmh*^-/-^ mice [4], contribute to the detrimental effects of Blmh depletion on Phf8, mTOR signaling, autophagy flux, and AβPP in N2a-APPswe cells.

### Blmh depletion by RNA interference increases H4K20me1 biding to the mTOR promoter in N2a-APPswe cells

**Fig. 4:**
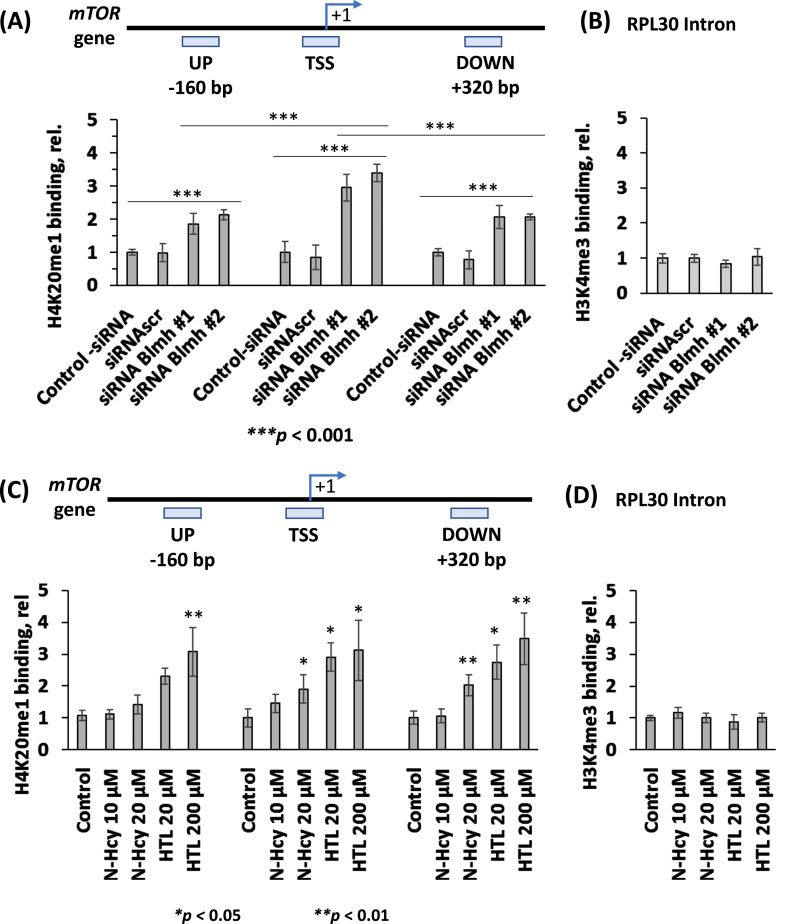
*Blmh* gene silencing or treatment with Hcy-thiolactone or *N*-Hcy-protein increases H4K20me1 binding at the *mTOR* promoter in mouse neuroblastoma N2a-APPswe cells. A) CUT&RUN assays with anti-H4K20me1 antibody show specific binding of H4K20me1 at the transcription start site (TSS) of the *mTOR* gene as well as downstream and upstream sites in Blmh siRNA-silenced N2a-APPswe cells. Bar graphs show the relative H4K20me1 binding at the indicated regions of the *mTOR* gene in N2a-APPswe cells transfected with two different siRNAs targeting the *Blmh* gene (siRNA *Blmh* #1 and #2). Transfections without siRNA (Control -siRNA) or with scrambled siRNA (siRNAscr) were used as controls. B) Control CUT&RUN experiment with anti-H3K4me3 antibody shows that *Blmh* gene-silencing did not affect the binding of H3K4me3 at the Rpl30 intron. RT-qPCR was conducted on the input and precipitated DNA fragments. Data are averages of three independent experiments. C) N2a-APPswe cells were treated with the indicated concentrations of *N*-Hcy-protein or Hcy-thiolactone (HTL) for 24 h at 37°C. Untreated cells were used as controls. The CUT&RUN assays with anti-H4K20me1 antibody show that H4K20me1 binds to the transcription start site (TSS) of the *mTOR* gene as well as downstream and upstream sites. Bar graphs show relative H4K20me1 binding at the indicated regions of the *mTOR* gene. D) A control CUT&RUN experiment with anti-H3K4me3 antibody shows that Hcy-thiolactone and *N*-Hcy-protein did not affect the binding of H3K4me3 at the Rpl30 intron. Panels C and D were reproduced with permission from [51]. RT-qPCR was conducted on the input and precipitated DNA fragments. Data are mean ± SD of three biologically independent experiments. *p*-values were calculated by one-way ANOVA with Tukey’s multiple comparisons test. *p<0.05, **p<0.01, ***p<0.001.

To find whether increased levels of the histone H4K20me1 mark can promote mTOR gene expression by binding to its promoter in Blmh-depleted cells, we conducted ChIP experiments using anti-H4K20me1 monoclonal antibody in the CUT&RUN assay (Fig. 4). The *Blmh* gene was silenced by transfecting N2a-APPswe cells with two different *Blmh*-targeting siRNAs (siRNA Blmh #1 and #2). The cells were permeabilized, treated with the anti-H4K20me1 antibody and a recombinant protein A/G-tagged micrococcal nuclease. DNA fragments released form N2a-APPswe cells were quantified by RT-qPCR using primers targeting the transcription start site (TSS) of the *mTOR* gene as well as upstream (UP) and downstream (DOWN) regions from the TSS.

We found that the binding of H4K20me1 was significantly increased at the mTOR TSS, mTOR UP, and mTOR DOWN sites in the *Blmh*-silenced N2a-APPswe cells compared to controls (Fig. 4A). Importantly, we found significantly more DNA fragments from the mTOR TSS compared with the mTOR UP and mTOR DOWN sites (Fig. 4A). Control experiments showed that binding of H3K4me3 to RPL30 intron was not affected by *Blmh* gene silencing (Fig. 4B). These findings show that Blmh depletion promoted H4K20me1 binding at the mTOR TSS site more efficiently than at the UP and DOWN sites.

The CUT&RUN experiments using anti-Phf8 antibody showed that Blmh depletion did not affect the binding of Phf8 to the mTOR gene (not shown).

### Hcy-thiolactone and N-Hcy-protein increase H4K20me1 binding to the mTOR promoter in N2a-APPswe cells

Because treatments with Hcy-thiolactone or *N*-Hcy-protein, metabolites that are elevated in Blmh-depleted mice [4], upregulated mTOR expression (Fig. 3C), it is likely that each of these metabolites can influence mTOR expression by promoting H4K20me1 binding at its promoter. To evaluate this contention, we conducted the CUT&RUN experiments in N2a-APPswe cells treated with Hcy-thiolactone or *N*-Hcy-protein using anti-H4K20me1 antibody and quantified the extent of H4K20me1 binding to the *mTOR* gene.

We found that Hcy-thiolactone significantly increased binding of H4K20me1 at the *mTOR* TSS site as well as at the UP, and DOWN sites [51] (Fig. 4C). *N*-Hcy-protein also significantly increased binding of H4K20me1 at the *mTOR* TSS and DOWN sites, but not at the UP site [51]. Control experiments show that the binding of H3K4me3 to RPL30 intron was not affected by treatments with Hcy-thiolactone or *N*-Hcy-protein [51] (Fig. 4D).

Binding of Phf8 at the *mTOR* TSS, UP and down sites was not affected by Hcy-thiolactone or *N*-Hcy-protein (not shown).

### Blmh depletion by RNA interference or treatments with Hcy-thiolactone and N-Hcy-protein promotes Aβ accumulation in N2a-APPswe cells

**Fig. 5:**
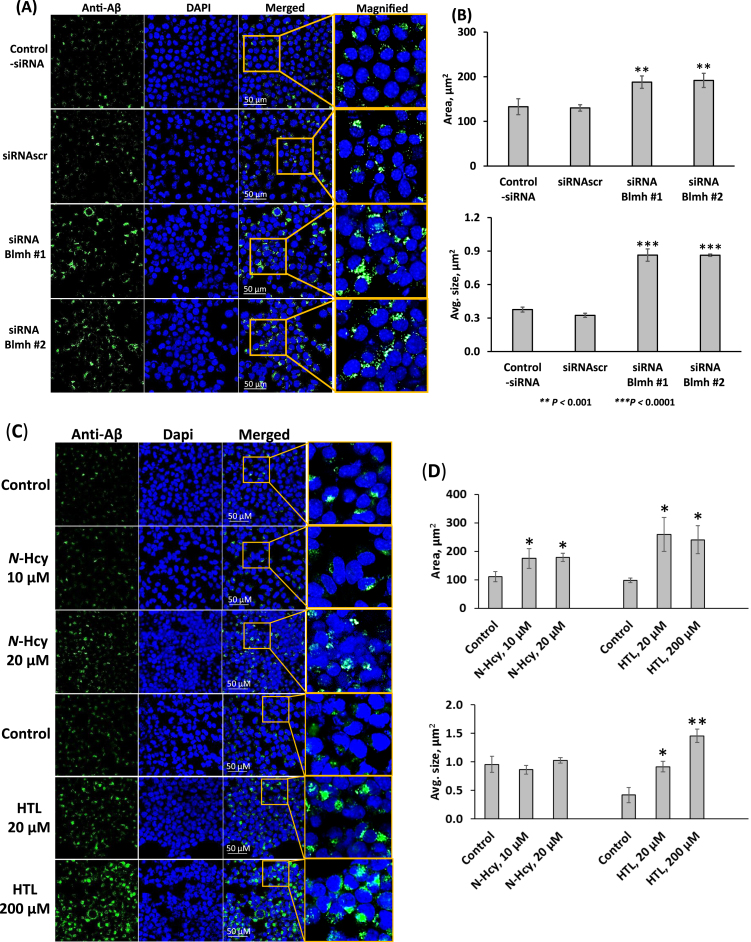
*Blmh* gene silencing or treatment with Hcy-thiolactone or *N*-Hcy-protein promotes Aβ accumulation in mouse neuroblastoma cells. Analysis of Aβ in mouse neuroblastoma N2a-APPswe cells was performed by confocal immunofluorescence microscopy using an anti-Aβ antibody. A, B) The cells were transfected with siRNAs targeting the *Blmh* gene (siRNA Blmh #1 and #2). Transfections without siRNA (Control -siRNA) or with scrambled siRNA (siRNAscr) were used as controls. Confocal microscopy images (A) are representative of at least *N* = 3 biologically independent experiments. Bar graphs (B) show the quantification of Aβ signals from *Blmh*-silenced and control cells. C, D) N2a-APPswe cells were treated with the indicated concentrations of *N*-Hcy-protein (N-Hcy) or Hcy-thiolactone (HTL) for 24 h at 37°C. Untreated cells were used as controls. Confocal microscopy images (C) are representative of at least *N* = 3 biologically independent experiments. Bar graphs (D) show the quantification of Aβ signals from cells treated with *N*-Hcy or HTL and untreated cells. Each data point is a mean ± SD of three biologically independent experiments with triplicate measurements in each. Panels (C) and (D) were reproduced with permission from [51]. *p*-values were calculated by one-way ANOVA with Tukey’s multiple comparisons test. *p<0.05, **p<0.001, ***p<0.0001.

To find whether Blmh depletion affects Aβ accumulation, we silenced the *Blmh* gene in N2a-APPswe cells by using RNA interference and quantified Aβ by fluorescence confocal microscopy. Towards this end, we transfected N2a-APPswe cells using two different *Blmh*-targeting siRNAs. The cells were permeabilized, treated with anti-Aβ antibody, Aβ was visualized with a fluorescent secondary antibody (Fig. 5A) and quantified (Fig. 5B). We found that the silencing of *Blmh* gene, which reduced Blmh protein levels by 80% (Supplementary Figure 2A), significantly increased the area and average size of fluorescent Aβ puncta in Blmh siRNA-treated cells compared to siRNAscr-treated cells or mock-treated cell without siRNA (Fig. 5B).

Because Blmh depletion elevates Hcy-thiolactone and *N*-Hcy-protein in mice [4], we next examined whether each of these metabolites can induce Aβ accumulation in N2a-APPswe cells. We found significantly increased area of fluorescent Aβ puncta in cells treated with Hcy-thiolactone or *N*-Hcy-protein, compared to untreated controls (Fig. 5C, D). However, while treatments with Hcy-thiolactone led to increased average size of the fluorescent Aβ puncta, treatments with *N*-Hcy-protein did not (Fig. 5C, D), suggesting different effects of these metabolites on the structure of Aβ aggregates. These findings suggest that Hcy-thiolactone and *N*-Hcy-protein promote accumulation of Aβ induced by Blmh depletion.

### Blmh gene deletion increases Aβ accumulation in brains of 5xFAD mice

**Fig. 6:**
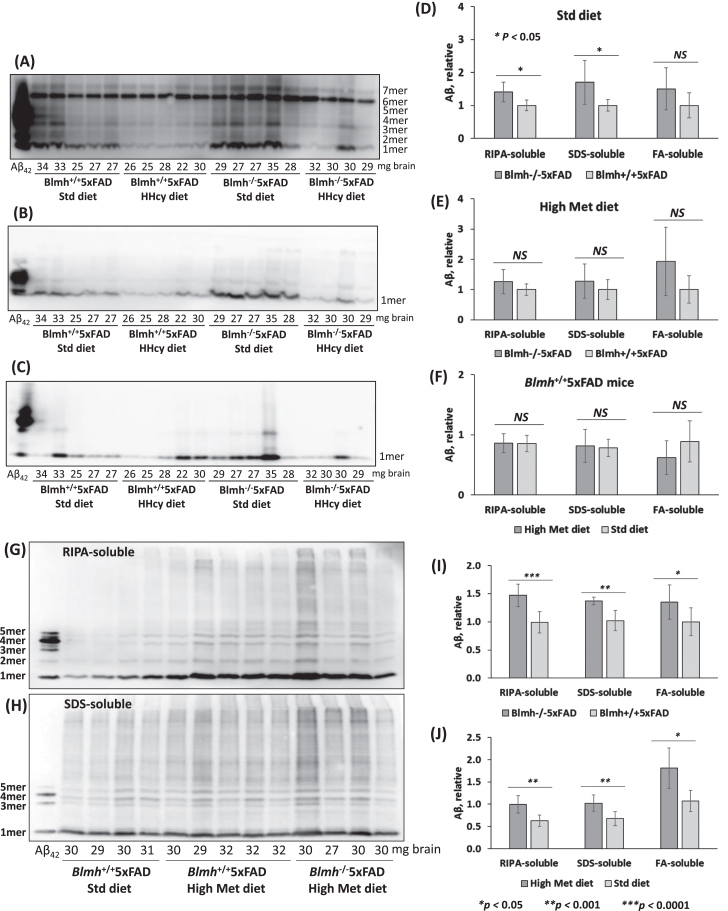
*Blmh* gene deletion promotes Aβ accumulation in 5xFAD mice. Analysis of Aβ in brains from 5-month-old (A-F) and 12-month-old (G-J) *Blmh*^-/-^5xFAD and *Blmh^+^*^/+^5xFAD mice fed with a standard or high Met diet (to induce HHcy) since weaning at the age of one month is shown. Brain extracts were analyzed on SDS-PAGE gels and Aβ was quantified by western blotting. Representative pictures of western blots of Aβ fractions extracted from brains with RIPA buffer (A, G), 2% SDS (B, H), and 70% formic acid (FA) (C) are shown. Numbers below each lane refer to the amount of brain (mg) from each mouse used in the experiment. A commercial Aβ_42_ standard is shown in the first lane from left in each blot. Total Aβ signals in each lane (standing for an individual mouse) were quantified by scanning all chemiluminescent bands. Bar graphs (D, E, F, I, L) show brain Aβ quantification for *Blmh*^-/-^5xFAD and *Blmh^+^*^/+^5xFAD mice (*n* = 8-10 mice/group). Data points for each mouse group represent mean ± SD with four independent measurements for each mouse. *p*-values were calculated by one-way ANOVA with Tukey’s multiple comparisons test. *p<0.05, **p<0.001, ***p<0.0001.

To examine if *Blmh* deletion can promote Aβ accumulation in the mouse brain, we generated *Blmh*^-/-^5xFAD mice and their *Blmh^+^*^/+^5xFAD siblings by crossing *Blmh*^-/-^ mice with Aβ-overproducing 5xFAD animals. We prepared SDS-soluble and FA-soluble Aβ fractions, which have most of the total Aβ [47], as well as an Aβ fraction extractable with a RIPA buffer, from brains of 5- and 12-month-old mice. Aβ was quantified in these brain extracts by western blotting using monoclonal anti-Aβ antibody. Representative western blots are shown in Fig. 6A, B, C, G, and H.

We found that RIPA- and SDS-soluble Aβ were significantly elevated in brains of 5-month-old *Blmh*^-/-^5xFAD mice compared to *Blmh^+^*^/+^5xFAD sibling controls in mice fed with a standard diet (Fig. 6D). The high Met diet abrogated effects of Blmh depletion on RIPA-soluble and SDS-soluble Aβ, while FA-soluble Aβ remained unaffected by Blmh depletion (Fig. 6E). The high Met diet did not affect RIPA-, SDS-, and FA-soluble Aβ in *Blmh^+^*^/+^5xFAD mice (Fig. 6F) and *Blmh*^-/-^5xFAD animals (quantification not shown).

In brains of 12-month-old *Blmh*^-/-^5xFAD mice, few with high Met diet, RIPA-, SDS-, and FA-soluble Aβ were significantly elevated compared with *Blmh^+^*^/+^5xFAD sibling controls fed with high Met diet (Fig. 6I). High Met diet significantly elevated RIPA-, SDS-, and FA-soluble Aβ in *Blmh^+^*^/+^5xFAD mice (Fig. 6J).

### Phf8 depletion upregulates Aβ but not AβPP in N2a-APPswe cells

The findings that Phf8 expression was significantly reduced in the brains of *Blmh*^-/-^ (Fig. 2A) and *Blmh*^-/-^5xFAD mice (Supplementary Figure 1A) and in the *Blmh*-silenced (Supplementary Figures 2B and 3B) or Hcy metabolite-treated (Fig. 3A) mouse neuroblastoma N2a-APPswe cells, suggested that Phf8 depletion by itself can affect biochemical pathways leading to Aβ accumulation. To examine this, we depleted Phf8 in N2a-APPswe cells by RNA interference and quantified by western blotting proteins that we found to be affected in the *Blmh*^-/-^ (Fig. 2) and *Blmh*^-/-^5xFAD (Supplementary Figure 1A) mouse brains.

Transfections with *Phf8*-targeting siRNAs reduced Phf8 protein levels in N2a-APPswe cells by 85% (Supplementary Figure 4A). Levels of H4K20me1 (Supplementary Figure 4B), mTOR (Supplementary Figure 4C), and pmTOR (Supplementary Figure 4D) were significantly upregulated in *Phf8*-silenced cells. Autophagy-related proteins Atg5 and Atg7 were significantly downregulated (Supplementary Figure 4E and F, respectively) while Becn1 was not affected in the *Phf8*-silenced cells (Supplementary Figure 4G). Notably, AβPP levels were not affected in the *Phf8*-silenced cells (Supplementary Figure 4H).

We also quantified Aβ by fluorescence confocal microscopy and found that Aβ was upregulated in the *Phf8*-silenced cells, manifested by significantly increased average size and signal intensity of fluorescent Aβ puncta compared to controls without siRNA or with siRNAscr (Supplementary Figure 4J, K). These findings show that upregulation of Aβ in *Phf8*-silenced cells was associated with impaired autophagy and not with the AβPP levels.

## DISCUSSION

Our present findings show that Blmh, a Hcy-thiolactone-hydrolyzing enzyme [3], has a protective role in the CNS. Specifically, we demonstrated that Blmh depletion, which causes accumulation of Hcy-thiolactone and *N*-Hcy-protein in mice [4], downregulated histone demethylase Phf8 and upregulated the H4K20me1 epigenetic mark. This increased H4K20me1 binding to the mTOR promoter and upregulated mTOR signaling, which in turn inhibited the autophagy flux. We also showed that Blmh depletion upregulated Aβpp and Aβ. These biochemical changes, also induced by Hcy-thiolactone an *N*-Hcy-protein in cultured neural cells, were associated with cognitive and neuromotor deficits in mice.

5xFAD mice accumulate high levels of Aβ beginning around 2 months of age [40] and develop cognitive impairments beginning at 4-5 months of age and sensorimotor impairments at about 9 months of age [52], performing worse than the wild type animals in the memory and sensorimotor tests (https://www.alzforum.org/research-models/5xfad-b6sjl). In the present work, we found that Blmh depletion aggravated those neurological impairments. Specifically, 1-year-old *Blmh*^-/-^5xFAD mice performed worse than *Blmh*^+/+^5xFAD animals in the NOR test (Fig. 1E), showing impaired memory, and in the hindlimb (Fig. 1F) and cylinder tests (Fig. 1G), showing sensorimotor impairments. We found similar memory and sensorimotor impairments also in 4-month-old *Blmh*^-/-^ *versus Blmh*^+/+^ mice (Fig. 1A-D), which do not accumulate Aβ. Our findings suggest that the absence of the Blmh protein is a dominant determinant that causes memory and sensorimotor impairments regardless of the presence or absence of the Aβ-generating transgene.

Memory and sensorimotor impairments in *Blmh*^-/-^ and *Blmh*^-/-^5xFAD mice can be caused, at least in part, by Phf8 depletion, which occurs in *Blmh*^-/-^ brains (Fig. 2A, Supplementary Figure 1A). Indeed, PHF8 depletion in humans is linked to intellectual disability, autism spectrum disorder, attention deficit hyperactivity disorder [32], and intellectual disability [33], while similar neurological deficits were found in *Phf8*^-/-^ mice [34]. However, Phf8 was not known to be associated with Aβ, a hallmark of AD. Our present findings that Phf8 depletion in mouse neuroblastoma cells, induced by *Phf8* gene silencing (Supplementary Figure 4A) or by supplementation with Hcy-thiolactone or *N*-Hcy-protein (Fig. 3A), significantly increased Aβ accumulation (Supplementary Figure 4J, K; Fig. 5C, D), suggest that Phf8 depletion can also underly the association of HHcy with AD [53].

In previous studies, we found that Blmh is a Hcy-thiolactone-hydrolyzing enzyme [3] and that Hcy-thiolactone and *N*-Hcy-protein are elevated in *Blmh*^-/-^ mice [4]. In the present study, we showed that treatments with Hcy-thiolactone or *N*-Hcy-protein mimicked the effects of Blmh depletion by RNA interference in mouse neuroblastoma cells. Specifically, Hcy-thiolactone/*N*-Hcy-protein *and* Blmh depletion downregulated Phf8 (Supplementary Figure 2B, Fig. 3A), elevated H4K20me1 (Supplementary Figure 2C, Fig. 3B), increased H4K20me1 binding at the mTOR promoter (Fig. 4A, C), and upregulated mTOR signaling (Supplementary Figure 2D, E; Fig. 3C, D), and affected autophagy-related proteins (Supplementary Figure 2G-M; Fig. 3E-G, 3I-L). These findings show that Blmh is a negative regulator of mTOR signaling by controlling levels of Hcy metabolites that affect the mTOR promoter occupancy by H4K20me1.

**Fig. 7:**
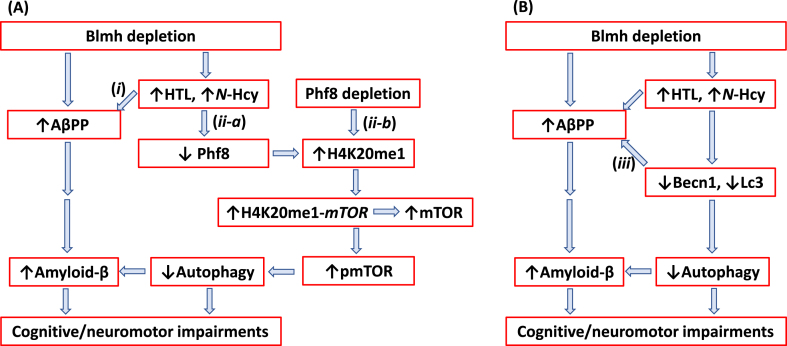
Hypothetical pathways leading to Aβ generation in *Blmh*^-/-^5xFAD mice. Panel A illustrates the AβPP (*i*) and Phf8 (*ii-a*) pathways. Panel B highlights the interaction (*iii*) between autophagy (Becn1) and AβPP pathways. Up and down arrows show direction of changes in the indicated variables. Blmh, bleomycin hydrolase; Hcy, homocysteine; HTL, Hcy-thiolactone; AβPP, amyloid-β protein precursor; mTOR, mammalian target of rapamycin; pmTOR, phospho-mTOR; Phf8, Plant Homeodomain Finger protein 8. See text for discussion.

Our findings also show that Phf8 is a mediator of Hcy-thiolactone/*N*-Hcy-protein effects on mTOR signaling, autophagy, and Aβ accumulation (Fig. 7). These findings directly link Hcy-thiolactone and *N*-Hcy-protein with dysregulated mTOR signaling (Fig. 3C, D) and its downstream outcomes such as impaired autophagy flux (Fig. 3I, J, K, L) and increased Aβ accumulation (Fig. 5) thereby providing a likely mechanism explaining neuropathy resulting from Blmh deficiency (Fig. 7) and accounting for the association of HHcy with AD [53]. This role of Phf8 is further supported by findings showing that *Phf8* gene silencing by RNA interference had the same effects on mTOR (Supplementary Figure 4C, D), autophagy (Supplementary Figure 4E-G), and Aβ (Supplementary Figure 4J, K) as did *Blmh* gene silencing (Supplementary Figure 2D, E, G-M, and Fig. 5A, B) or the treatments with Hcy-thiolactone or *N*-Hcy-protein (Figs. 3 and 5C and 5D).

In the present study we showed that *Blmh* gene deletion led to Aβpp upregulation in *Blmh*^-/-^ (Fig. 2 H) and AβPP in *Blmh*^-/-^5xFAD mice (Supplementary Figure 1I). These findings were recapitulated in mouse neuroblastoma cells by *Blmh* silencing, which upregulated *AβPP* gene expression at the protein (Supplementary Figure 2F) and mRNA levels (Supplementary Figure 3D). In contrast, *Phf8* gene silencing did not affect AβPP expression (Supplementary Figure 4 H). These findings suggest that Blmh and AβPP proteins interact with each other in the CNS while Phf8 does not interact with AβPP. The Blmh-AβPP interaction is most likely direct, as suggested by findings of other investigators. For example, one study has shown that human BLMH interacts with AβPP *in vitro* and that overexpressed BLMH has the ability to process human AβPP to Aβ in the 293-HEK and CHO cells [21]. Another study reported that rat Blmh can further hydrolyze Aβ *in vitro*, with fibrillar Aβ_40_ and Aβ_42_ being more resistant than nonfibrillar peptides [22]. Alternatively, BLMH can regulate mTOR expression via binding to the *mTOR* promoter. This possibility is supported by findings showing that BLMH binds to DNA [54, 55]. However, the mechanism underlying the regulation of AβPP by Blmh needs to be elucidated in future studies.

We found that Blmh depletion downregulated Phf8 in mouse brain (Fig. 2A, Supplementary Figure 1A) and mouse neuroblastoma cells (Supplementary Figures 2B and 3B), upregulated AβPP in mouse brain (Fig. 2H, Supplementary Figure 1I) and mouse neuroblastoma cells (Supplementary Figures 2F and 3D), and Aβ in mouse brain (Fig. 6D-E, I-J) and mouse neuroblastoma cells (Fig. 5A, B). In contrast, Phf8 depletion had no effect on AβPP (Supplementary Figure 4H, I) but still upregulated Aβ (Supplementary Figure 4J, K). These findings suggest that Aβ accumulation in Blmh-depleted mouse brains can occur via three pathways shown in Fig. 7.

In pathway (*i*) (Fig. 7A) Hcy metabolites upregulate AβPP in Blmh-depleted (Supplementary Figure 2F) or Hcy-thiolactone/*N*-Hcy-protein-treated mouse neural cells (Fig. 2H). In pathway (*ii-a*) (Fig. 7A), Hcy metabolites downregulate Phf8, which leads to reduced autophagy flux (Fig. 3I-K; Supplementary Figure 2J-L) resulting in Aβ accumulation due to impaired clearance. Direct depletion of Phf8 by RNA interference also starts a similar pathway (*ii-b*) (Fig. 7A) that leads to Aβ accumulation via impaired autophagy (Supplementary Figure 4). However, these pathways need to be confirmed in future experiments involving Phf8 overexpression or mTOR knockdown (by RNA interference or pharmacological inhibition with rapamycin) in *Blmh*-silenced cells.

Our findings that AβPP upregulation (Fig. 2H, Supplementary Figure 1I) was associated with Becn1 downregulation in brains of *Blmh*^-/-^ (Fig. 2E) and *Blmh*^-/-^5xFAD mice (Supplementary Figure 1E) as well as in N2a-APPswe mouse neuroblastoma cells (Supplementary Figure 2F, G; Supplementary Figure 3D, G) suggest another pathway ((*iii*) in Fig. 7B) leading to Aβ accumulation. Pathway (*iii*) involves the interaction between Bcln1 and AβPP in which Becn1 is a negative regulator of AβPP expression and processing (Fig. 7B). Becn1, a protein with a key role in the initiation of autophagy, is known to decrease in human AD brains while genetic reduction of Becn1 in transgenic mice that overexpress AβPP (*AβPP*^+^*Becn*^+/-^ mice) increased Aβ accumulation in neuronal cells [38]. Becn1 was also shown to regulate AβPP processing and turnover. Specifically, depletion of Bcln1 by RNA interference in rat neuroblastoma cells expressing human AβPP transgene (B103/hAPPwt cells) increased AβPP, Lc3, and Aβ, while Becn1 overexpression reduced AβPP levels [37]. The involvement of autophagy in Blmh depletion-induced AβPP and Aβ accumulation needs to be confirmed in future experiments by boosting autophagy (e.g., by treatment with TAT-Beclin1), which should rescue AβPP and Aβ accumulation in Blmh-depleted cells.

Notably, we found that HHcy induced by a high Met diet *and Blmh* gene deletion caused similar changes in the Phf8->H4K20me1->mTOR->autophagy pathway (Fig. 2, Supplementary Figure 1) and Aβ accumulation (Fig. 6I, J). These findings can be explained by our earlier findings showing that a common primary biochemical outcome of the *Blmh* gene deletion and high Met diet was the same: accumulation of Hcy-thiolactone and *N*-Hcy-protein [4, 56]. Indeed, as we also found in the present study, treatments with Hcy-thiolactone or *N*-Hcy-protein induce changes in the Phf8->H4K20me1->mTOR->autophagy pathway and Aβ accumulation in mouse neuroblastoma cells (Figs. 3 and 5C, 5D) like those seen in *Blmh*^-/-^ mice or mice fed with high Met diet (Fig. 2, Supplementary Figure 1, Fig. 6I, J). These findings also suggest that dysregulation of Hcy metabolism in general would affect the Phf8->H4K20me1->mTOR->autophagy pathway and the central nervous system. Indeed, homocysteine metabolites inhibit autophagy, elevate Aβ, and induce neuropathy by impairing Phf8/H4K20me1-dependent epigenetic regulation of mTOR in cystathionine β-synthase-deficient mice [57].

Notably, as shown in the present work, Blmh deficiency or HHcy induced by a high Met diet increased H4K20me1 methylation levels due to downregulation of the histone demethylase Phf8 (Fig. 2B, Supplementary Figure 1B). HHcy is also known to affect DNA and protein methylation via *S*-adenosylhomocysteine (AdoHcy, an inhibitor of cellular AdoMet-dependent methylation reactions), which underlies the pathology of HHcy-associated human disease (reviewed in [58]). However, possible inhibition of H4K20 methylase by AdoHcy would have an opposing effect, i.e., would reduce H4K20me1 levels. The present findings, linking Blmh with the status of the histone H4K20me1 methylation, are reminiscent of our recent findings showing that Pon1 deletion in mice elevated the H4K20me1 methylation levels via downregulation of Phf8 [51]. Thus, these two Hcy-thiolactone-detoxifying enzymes exert similar effects on H4K20me1 levels. Although there is no evidence that Blmh or Pon1 are linked to DNA methylation, our present and earlier findings supply the first evidence that Blmh and Pon1 can affect histone methylation status.

In conclusion, our findings show that Blmh interacts with AβPP and the Phf8/H4K20me1/mTOR/autophagy pathway, and that disruption of these interactions leads to Aβ accumulation and cognitive and neuromotor deficits. By revealing the mechanism by which Blmh prevents Aβ generation and cognitive/neuromotor deficits, the hallmarks of AD, our findings significantly expand our understanding of how Blmh maintains brain homeostasis.

## Supplementary Material

Supplementary Material

## Data Availability

The data that support the findings of this study are available in the methods and/or supplementary material of this article.
