# Supplementary material for: Deletion of the Homocysteine Thiolactone Detoxifying Enzyme Bleomycin Hydrolase, in Mice, Causes Memory and Neurological Deficits and Worsens Alzheimer’s Disease-Related Behavioral and Biochemical Traits in the 5xFAD Model of Alzheimer’s Disease
Source: J Alzheimers Dis. 2023 Oct 10;95(4):1735–55. doi: 10.3233/JAD-230578 (PMC10578231; doi:10.3233/JAD-230578)
Supplement: Supplementary Material [file jad-95-jad230578-s001.pdf]

Supplementary Material

Deletion of the Homocysteine Thiolactone Detoxifying Enzyme Bleomycin Hydrolase, in Mice, Causes Memory and Neurological Deficits and Worsens Alzheimer's Disease-Related Behavioral and Biochemical Traits in the 5xFAD Model of Alzheimer's Disease

Supplementary Figure 1. Blmh depletion affects the expression of histone demethylase Phf8, histone H4K20me1 epigenetic mark, mTOR signaling, autophagy, and AβPP in the *Blmh*$^{-/-}$ 5xFAD mouse brain.

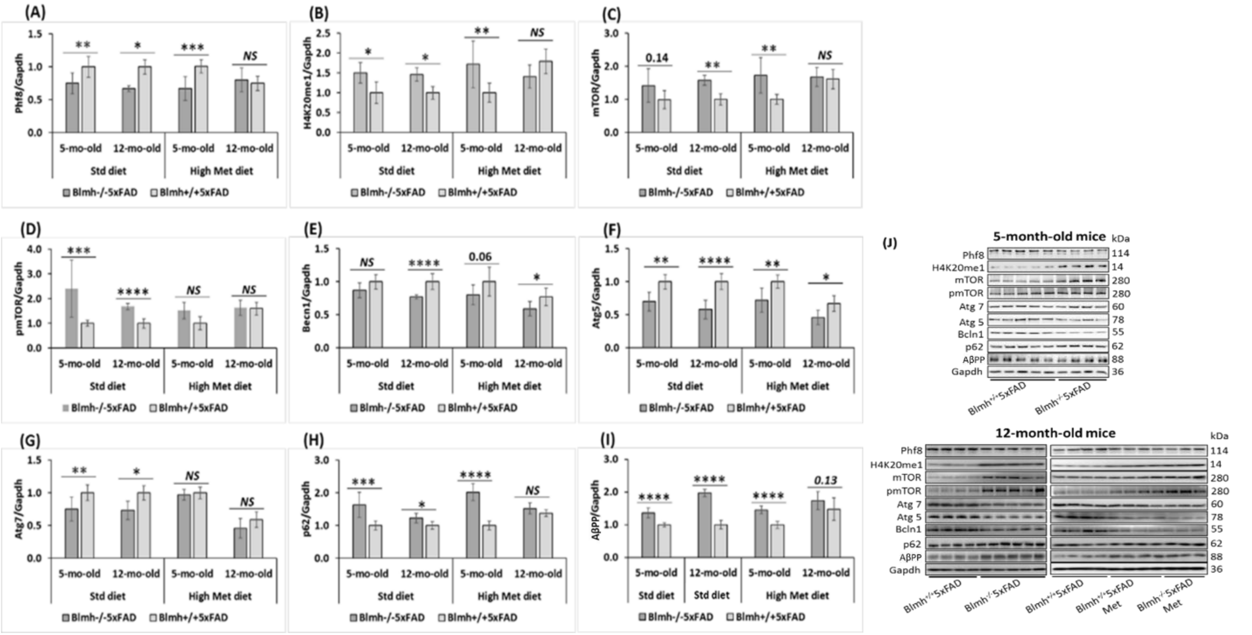

One-month-old *Blmh*$^{-/-}$5xFAD mice and *Blmh*$^{+/+}$5xFAD sibling controls fed with HHcy high Met diet (1% Met in drinking water) or control diet for 4 and 11 months were used in experiments. Each group included 7-10 mice of both sexes. Bar graphs illustrating quantification of the following brain proteins by western blotting are shown: Phf8 (A), H4K20me1 (B), mTOR (C), pmTOR (D), Bcln1 (E), Atg5 (F), Atg7 (G), p62 (H), and AβPP (I). Gapdh protein was used as references for normalization. Panel (J) shows representative pictures of western blots. Data are mean ± SD values of three independent experiments. Numerical values in panels (E) and (I) show p values > 0.05 < 0.015. p values were calculated by one-way ANOVA with Tukey's multiple comparisons test. $^{*}p < 0.05$, $^{**}p < 0.01$, $^{***}p < 0.001$, $^{****}p < 0.0001$.

Supplementary Figure 2. *Blmh* gene silencing in mouse neuroblastoma N2a-APPswe cells recapitulates changes in histone demethylase Phf8, H4K20me1, mTOR signaling, AβPP, and autophagy-related protein levels seen in *Blmh*$^{-/-}$ mouse brain.

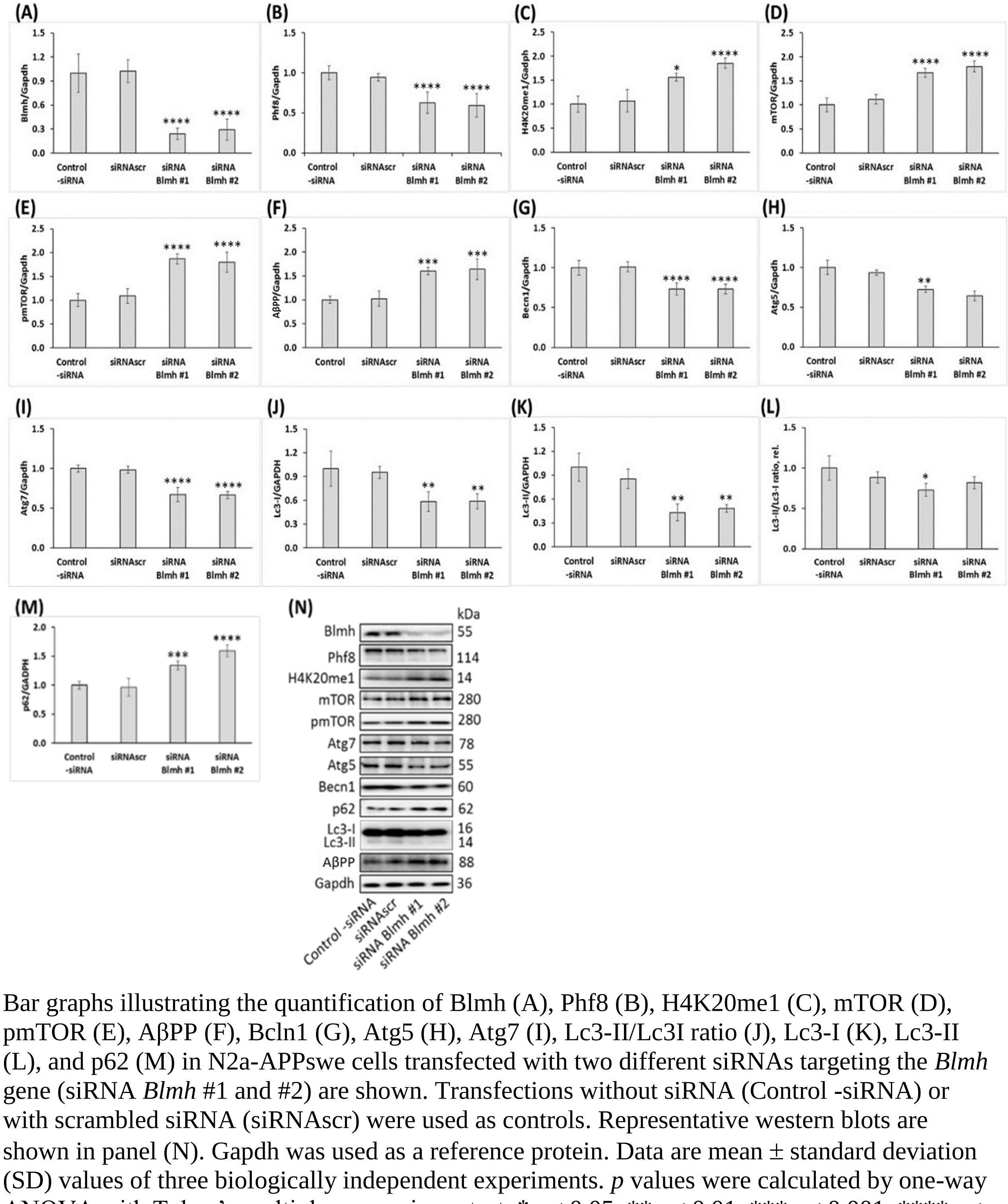

Bar graphs illustrating the quantification of Blmh (A), Phf8 (B), H4K20me1 (C), mTOR (D), pmTOR (E), AβPP (F), Bcln1 (G), Atg5 (H), Atg7 (I), Lc3-II/Lc3I ratio (J), Lc3-I (K), Lc3-II (L), and p62 (M) in N2a-APPswe cells transfected with two different siRNAs targeting the *Blmh* gene (siRNA *Blmh* #1 and #2) are shown. Transfections without siRNA (Control -siRNA) or with scrambled siRNA (siRNAscr) were used as controls. Representative western blots are shown in panel (N). Gapdh was used as a reference protein. Data are mean ± standard deviation (SD) values of three biologically independent experiments. *p* values were calculated by one-way ANOVA with Tukey's multiple comparisons test. *$p < 0.05$, **$p < 0.01$, ***$p < 0.001$, ****$p < 0.0001$ versus 'control -siRNA' plus 'siRNAscr'.

Supplementary Figure 3. *Blmh* gene silencing affects expression of mRNAs for Phf8, mTOR, AβPP, and autophagy-related proteins in mouse neuroblastoma N2a-APPswe cells.

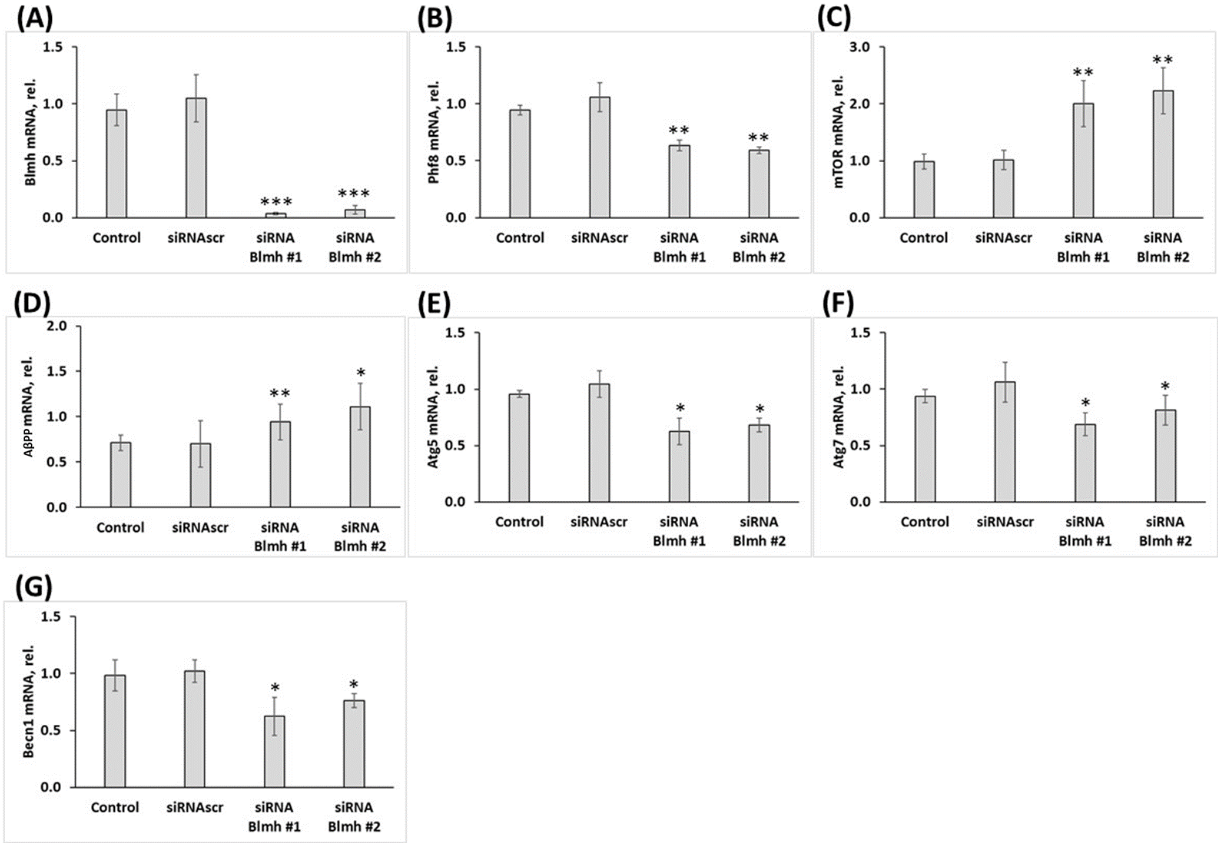

Bar graphs illustrating the quantification by RT-qPCR of mRNAs for Blmh (A), Phf8 (B), mTOR (C), AβPP (D), Atg5 (E), Atg7 (F), and Bcln1 (G), in N2a-APPswe cells transfected with two different siRNAs targeting the *Blmh* gene (siRNA Blmh #1 and #2) are shown. Gapdh mRNA was used as a reference. Transfections without siRNA (Control) or with scrambled siRNA (siRNAscr) were used as controls. Data are mean ± standard deviation (SD) values from three biologically independent experiments. p values were calculated by one-way ANOVA with Tukey's multiple comparisons test. $^*p < 0.01$, $^{**}p < 0.001$, or $^{***}p < 0.001$ versus 'control -siRNA' plus 'siRNAscr.'

Supplementary Figure 4. Phf8 depletion promotes Aβ accumulation mediated by upregulation of mTOR signaling and inhibition of autophagy in the mouse neuroblastoma N2a-APPswe cells.

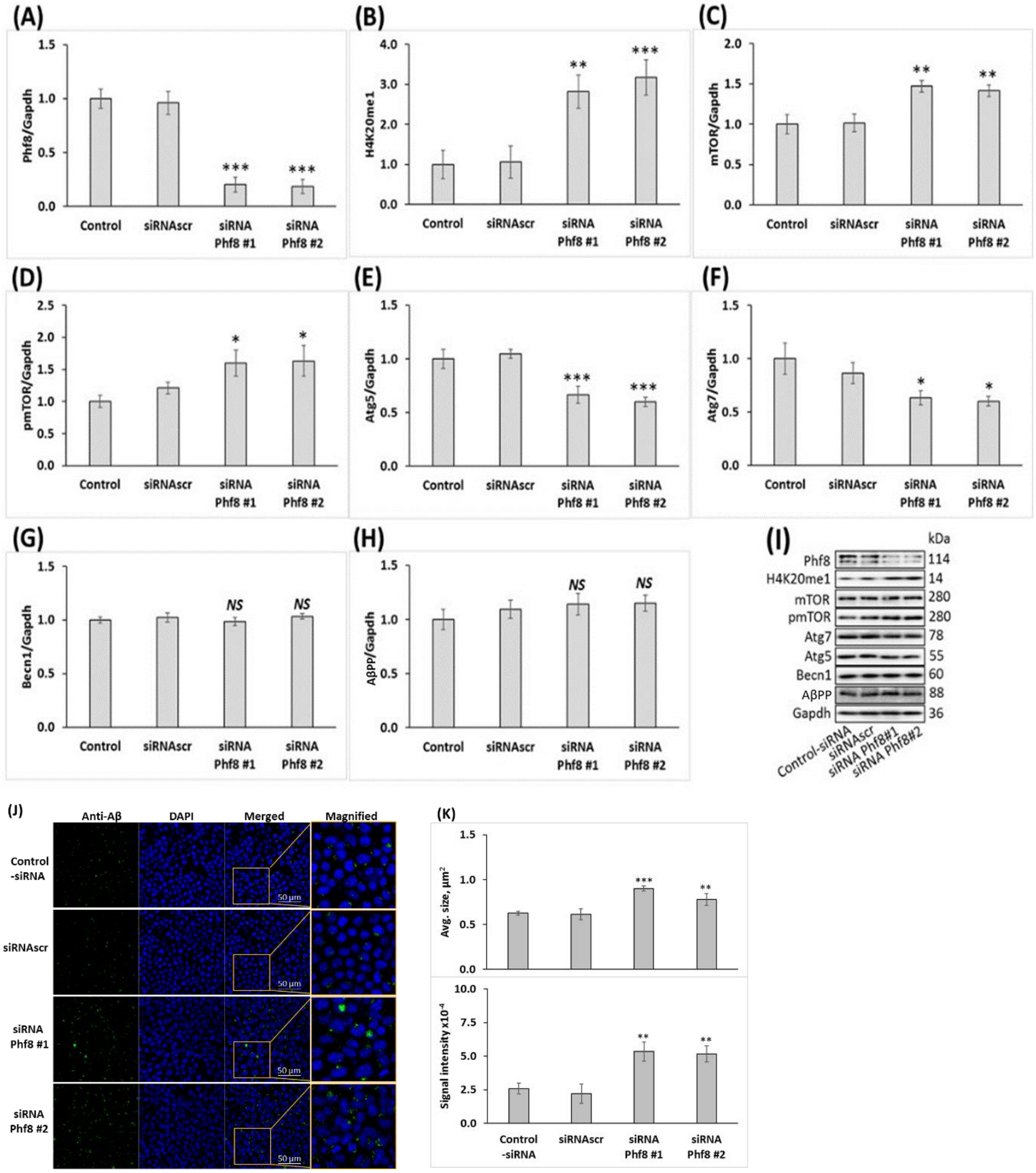

The cells were transfected with siRNAs targeting the *Phf8* gene (Phf8 siRNA #1 and #2). Transfections without siRNA (Control -siRNA) or with scrambled siRNA (siRNAscr) were used as controls. Proteins were quantified by western blotting. Bar graphs illustrate levels of (A) Phf8, (B) H4K20me1, (C) mTOR, (D) pmTOR, (E) Atg5, (F) Atg7, (G) Bcln1, and (H) AβPP. Aβ was detected and quantified by confocal immunofluorescence microscopy using anti-Aβ antibody. (I) Confocal microscopy images of Aβ signals from *Phf8*-silenced and control N2a-APPswe cells. (J) Bar graphs show quantification of Aβ signals. Data are mean ± standard deviation (SD) values from three biologically independent experiments. p values were calculated by one-way ANOVA with Tukey's multiple comparisons test. *p < 0.01, **p < 0.001, or ***p < 0.001 versus 'control - siRNA' plus 'siRNAscr.' *NS*, not significant.

Supplementary Table 1. Primers used for PCR or RT-qPCR

Gene	Primer sequence
APP<br>*App*	Forward: 5′-CTTCCCCAAGATCCTGATAAACT-3′
	Reverse: 5′-CCGGGTGTCTCCAGGTACT-3′
Atg5	Forward: 5′-AAGGCACACCCCTGAAATGG-3′
	Reverse: 5′-TGATGTTCCAAGGAAGAGCTGAA-3′
Atg7	Forward: 5′-GCCAACTCCACACTGCTTTC-3′
	Reverse: 5′-TCTTCTGGGTCAGTTCGTGC-3′
Actb	Forward: 5′-GCAGGAGTACGATGAGTCCG-3′
	Reverse: 5′-ACGCAGCTCAGTAACAGTCC-3′
Becn1	Forward: 5′-GAGGAAGCTCAGTACCAG CG-3′
Blmh	Reverse: 5′-CCAGATGTGGAAGGTGGCAT-3′
	Forward p1: 5′-CACTGTAGCTGTACTCACAC-3’
	Reverse p2: 5′-GCGACAGAGTACCATGTAGG-3′ (exon 3);<br>Reverse p3: 5′-ATTTGTCACGTCCTGCACGACG-3′ (neomycin cassette)
hAPP transgene in *5xFAD* mice	Forward: 5′-AGAGTACCAACTTGCATGACTACG-3′;
	Reverse: 5′-ATGCTGGATAACTGCCTTCTTATC-3′
hPS1 transgene in *5xFAD* mice	Forward: 5′-GCTTTTTCCAGCTCTCATTTACTC-3′
	Reverse: 5′-AAAATTGATGGAATGCTAATTGGT-3′
Gapdh	Forward: 5′-GGACTGGATAAGCAGGGCG-3′
	Reverse: 5′-TTTTGTCTACGGGACGAGGC-3′
mTOR	Forward: 5′-GCCACTCTCTGACCCAGTTC-3′
	Reverse: 5′-ATGCCAAGACACAGTAGCGG-3′
Phf8	Forward: 5′-TGGGAGCATGCTTCAAGG-3′
	Reverse: 5′-GATTTCAAAGCAGGGTCATCA-3′
mTOR upstream TSS	Forward: 5′-TTGCCAACTGGTGCTCGTTT-3′
	Reverse: 5′-AAGAATTGGAGCTCGGGACC-3′
mTOR TSS	Forward: 5′-GGATGTTCCTCCCCAATCTTCG-3′
	Reverse: 5′-CAGACCCACCTAACTGACCGT-3′
mTOR downstream TSS	Forward: 5′-TAGGGGGCAGATCCCGAAAC-3′
	Reverse: 5′-CACTGTAGCTGTAACTCACAC-3′

TSS, transcription start site
